# Supplementary material for: ABCD1 Transporter Deficiency Results in Altered Cholesterol Homeostasis
Source: Biomolecules. 2023 Aug 31;13(9):1333. doi: 10.3390/biom13091333 (PMC10526550; doi:10.3390/biom13091333)
Supplement: Supplementary file 1 [file biomolecules-13-01333-s001.zip › biomolecules-2570104-supplementary.pdf]

Supplementary Materials

ABCD1 Transporter Deficiency Results in Altered Cholesterol Homeostasis

Agnieszka Buda, Sonja Forss-Petter, Rong Hua, Yorrick Jaspers, Mark Lassnig,
Petra Waidhofer-Söllner, Stephan Kemp, Peter Kim, Isabelle Weinhofer and Johannes Berger

List of supplementary materials:

1. Supplementary Table S1. Primers used in RT-qPCR gene expression analysis.
2. Supplementary Table S2. Raw data from the lipidomic analysis of cholesterol ester-fatty acid species in human control and X-ALD fibroblasts.
3. Supplementary Table S3. Raw data from the lipidomic analysis of cholesterol ester-fatty acid species in the brain tissue of WT and *Abcd1* KO mice.
4. Supplementary Table S4. Raw data from the lipidomic analysis of cholesterol ester-fatty acid species in the spinal cord tissue of WT and *Abcd1* KO mice.
5. Supplementary Table S5. *P*-values of *t*-tests comparing the effect of cholesterol condition on gene expression within the control and X-ALD genotype groups.
6. Supplementary Figure S1. Lipidomic analysis of cholesterol ester-fatty acid species in human control and X-ALD fibroblasts and in the CNS of WT and *Abcd1* KO mice.
7. Supplementary Figure S2. Lipidomic analysis of triglyceride-fatty acid species in human control and X-ALD fibroblasts.
8. Supplementary Figure S3. Cholesterol triggers proper regulation of the SREBP2 pathway in control and X-ALD fibroblasts at the mRNA level.
9. Supplementary Figure S4. Dysregulated cholesterol-associated gene expression in X-ALD is aggravated by combined cholesterol and 25-HC exposure.
10. Supplementary Figure S5. LXR agonist TO901317 does not reduce the levels of the most significantly accumulating CE-VLCFA species, CE(26:0) and CE(26:1), in the CNS of X-ALD mice.
11. Supplementary Figure S6. Increased induction of LDs is manifest in X-ALD fibroblasts already after 24 h of cholesterol loading.
12. Supplementary Figure S7. ABCD1 protein levels in various X-ALD-derived fibroblast lines.
13. Supplementary Figure S8. Progesterone triggers LD lipolysis in fibroblasts without affecting the expression of genes associated with the cortisol pathway.

Supplementary Table S1

Supplementary Table S1. Primers used in RT-qPCR gene expression analysis

Gene	Primer sequence
**Human**	
ABCA1	Forward: 5´-tgtctgggatatgtgcaattacg-3´<br>Reverse: 5´gcttattgtcggtgaacagct-3´
HMGCR	Forward: 5´-acctttccagagcaagcaca-3´<br>Reverse: 5´ctgacgtacccctgacatgg-3´
HPRT	Forward: 5´-ccctggcgtcgtgattagt-3´<br>Reverse: 5´-caggtcagcaaagaatttatagcc-3´
LDLR	Forward: 5´-gaggaaaactgcgctgtgg-3´<br>Reverse: 5´-tgtcacattaacgcagccaac-3´
NCEH1	Forward: 5´-tgcccttggacaacagtttact-3´<br>Reverse: 5´ttcaccatgacatagcgggg-3´
PLIN2	Forward: 5´-atcagccatcaactcagattgtt-3´<br>Reverse: 5´-ggtcttgtccatcacccctg- 3´
SOAT1	Forward: 5´-atcttgccaggtgtgctgat-3´<br>Reverse: 5´-acatcctgtcaccaaagcgt-3´
SREBP2	Forward: 5´-tgcccttcaagtaccaaccc-3´<br>Reverse: 5´-ggttgtccgcctttctcctt-3´
**Mouse**	
Abca1	Forward: 5´-tgaagccagttgtgacaaaac-3´<br>Reverse: 5´-agaaacatcacctcctgccg-3´
Apoe	Forward: 5´-tgtttcggaaggagctgact-3´<br>Reverse: 5´-tgtgtgacttgggagctctg-3´
Hmgcr	Forward: 5´-caccatgtcaggcgtccg-3´<br>Reverse: 5´-ctaggaccagcgacacacag-3´
Hprt	Forward: 5´-acttcagggatttgaatcacgtt-3´<br>Reverse: 5´-gcagatggccacaggactaga-3´
Soat1	Forward: 5´-cgagacaactacccaaggactc-3´<br>Reverse: 5´-acatcctgtcaccaaagcgt-3´

Supplementary Table S2

Supplementary Table S2. Raw data from the lipidomic analysis of cholesterol ester-fatty acid species in human control and X-ALD fibroblasts.

Phenotype	Control 1	Control 2	Control 3	Control 4	Control 5	Control 6	Control 7	Control 8	X-ALD 1	X-ALD 2	X-ALD 3	X-ALD 4	X-ALD 5	X-ALD 6	X-ALD 7	X-ALD 8
**Metabolite**																
**CE(16:0)**	640.8370	675.3150	387.4170	360.1410	1048.9500	2130.4300	891.0750	972.3110	247.7140	695.7330	701.1130	667.7050	809.4210	795.8980	1244.4700	334.9710
**CE(16:1)**	2213.7300	2189.5000	1432.4900	1234.5500	3408.4100	6243.6500	2563.4700	3047.5200	948.2620	2439.1600	2831.5400	2494.8600	2420.9400	2629.3300	3217.5100	1321.2300
**CE(16:2)**	92.2952	112.3510	69.3092	58.6393	160.0360	259.2070	127.0430	145.8060	36.9689	124.0850	138.8870	106.5510	124.9670	130.1630	164.6540	63.6472
**CE(16:3)**	83.8159	80.5740	37.0466	37.2292	83.4516	165.3760	64.2267	80.3569	30.1614	86.1458	95.2567	79.9353	104.5370	90.9781	111.8140	31.7312
**CE(17:0)**	367.1850	312.7030	255.1140	231.1490	383.2670	709.2730	432.5440	569.2600	192.2530	313.3330	322.3580	293.9900	414.5410	436.8240	444.9440	221.8470
**CE(17:1)**	1097.0000	984.2310	625.5160	605.8420	1553.8800	2910.0800	1246.4400	1618.5300	439.2370	1061.4500	1242.5800	997.5810	1278.1400	1062.4200	1749.4000	551.5000
**CE(17:2)**	109.8490	54.6171	59.8684	39.0513	131.6810	209.1410	99.8090	117.7200	35.9395	99.8260	131.5370	83.8718	111.1450	146.8770	164.5330	30.9482
**CE(18:0)**	132.0760	151.8620	74.3391	72.2870	274.4930	527.9500	139.2000	178.2420	41.9006	160.7320	244.2920	186.6460	234.6690	208.0900	318.7790	71.7513
**CE(18:1)**	22075.1000	22739.6000	12136.8000	11774.2000	30637.7000	58492.6000	25702.8000	29603.5000	7744.6700	23622.9000	24758.8000	25481.3000	31030.1000	28342.6000	34929.5000	11490.0000
**CE(18:2)**	10280.9000	10274.5000	6013.6700	5477.9700	15423.3000	29308.0000	10916.4000	13473.6000	3782.0900	12252.8000	12440.0000	12771.6000	15021.5000	11583.6000	18259.5000	5855.7000
**CE(18:3)**	1855.4600	1936.2700	1134.8600	1100.1700	2579.0400	5165.9500	2220.8400	2202.6000	759.8430	2180.4400	1471.5100	2349.5800	2707.7500	2464.2200	3136.1700	1118.3300
**CE(18:4)**	57.6727	54.0865	19.5234	16.0045	52.4509	108.1130	39.9797	24.9138	15.5482	66.1838	108.2850	52.8845	102.6110	111.1440	70.8919	23.5669
**CE(19:0)**	102.5190	107.6280	66.7947	55.8178	159.5650	196.0830	110.3840	136.2400	38.5591	120.8860	74.7994	133.0870	154.8470	174.7220	195.0690	61.1650
**CE(19:1)**	2741.1800	2565.8100	1788.4200	1750.4100	2844.9000	5149.2600	3160.7200	4214.3400	1257.4200	2193.1600	2863.0800	2394.4600	3662.6300	3690.9300	3191.1500	1521.1000
**CE(19:2)**	1316.5100	1245.3100	730.5540	653.0880	1530.9200	2944.7900	1299.8900	1766.5100	468.6830	1218.0200	1525.5100	1388.8500	1787.4600	1733.3600	1855.3300	634.4800
**CE(19:3)**	339.5230	284.2060	187.2540	156.9330	266.2360	518.8230	307.4560	391.3860	114.1750	308.1280	445.3570	380.1330	509.1600	514.3100	487.9460	149.9270
**CE(20:1)**	332.4090	398.5960	146.4510	124.4050	398.8980	432.8040	301.2730	309.1760	100.4320	481.8440	422.6690	732.8010	840.1730	608.1200	543.9850	160.0760
**CE(20:2)**	1168.7100	1192.4800	478.2410	477.8170	721.3690	1392.3400	883.5960	949.6360	321.0870	1068.4700	2143.4100	2870.7400	3316.4800	1824.7600	1035.0200	452.6440
**CE(20:3)**	4628.7100	4852.4700	2031.5400	2017.8400	3471.5000	6855.7000	3585.0900	3266.6200	1128.4000	3733.4500	6848.6000	8314.4500	9958.3100	8565.4000	4337.4300	1777.6500
**CE(20:4)**	15045.0000	15846.7000	9675.6400	9077.1100	19572.1000	36876.4000	16204.0000	18998.6000	6027.0600	16100.1000	18792.6000	19464.3000	22802.4000	22998.9000	24130.2000	8842.3600
**CE(20:5)**	4767.2200	5141.6400	2270.5800	2218.5900	3507.2900	7383.0100	4181.0900	3979.7900	1398.5200	3550.0300	6690.9800	6012.7600	8277.9300	12339.4000	4925.9400	2119.3800
**CE(21:3)**	612.3370	624.0660	274.1590	280.0820	426.2060	813.2460	437.7100	487.4080	172.2650	423.6340	799.2390	873.7130	1262.9100	1186.2400	529.1300	241.1870
**CE(21:4)**	910.1130	877.5820	545.9960	496.4260	981.5320	1599.2400	936.8900	1432.9700	368.6470	638.6850	776.5490	788.2420	1131.8900	1370.7700	1262.7000	427.9620
**CE(21:5)**	368.5470	385.7360	177.1910	148.9390	268.9820	468.4860	328.7460	403.9310	130.6980	231.5310	550.1310	371.9900	529.0700	1081.4300	353.7020	142.1940
**CE(22:1)**	35.0068	42.3350	15.1288	14.8182	13.0149	29.8120	32.5092	31.6573	13.6065	57.9386	107.4100	123.0810	148.1410	81.1875	27.6727	16.1777
**CE(22:2)**	87.9056	116.8900	34.5729	39.8173	44.3055	46.6524	61.0031	67.1814	24.9084	111.0620	255.2180	389.7250	518.7280	209.3730	67.9616	37.5278
**CE(22:3)**	327.7750	364.2920	140.8190	146.1140	136.8490	311.6890	192.8170	233.0540	76.9061	296.2230	732.7040	1202.8100	1573.1000	842.1900	213.1630	106.8590
**CE(22:4)**	1622.1100	1878.3700	885.4320	890.2350	626.4330	1236.7100	1079.4800	850.4520	283.9480	1003.1700	2684.2900	4145.8800	5323.9100	4214.7800	986.1300	447.6820
**CE(22:5)**	8477.6000	9358.2900	3659.0300	3283.2200	3858.7200	7687.4300	5745.2100	4643.1000	1807.9100	6144.9600	10737.0000	15459.7000	19252.1000	14864.1000	3581.0000	2510.9900
**CE(22:6)**	19282.4000	20955.2000	8909.4700	8311.5000	15917.9000	32178.9000	18430.6000	17779.1000	5378.7700	18298.0000	27613.0000	37628.4000	46863.7000	38027.9000	20503.2000	7865.1000
**CE(23:3)**	50.1736	57.7956	19.4002	18.1300	24.1729	56.4219	31.3338	39.5737	7.8896	36.3444	108.5130	159.0940	185.5490	117.5660	26.8220	16.4646
**CE(24:1)**	111.4690	139.5220	39.5155	38.0844	7.1206	74.4860	67.5318	80.5032	35.2591	169.0380	451.5310	344.3780	393.7840	293.8470	8.3021	45.7704
**CE(24:2)**	46.9004	60.7257	18.5217	21.0977	8.5731	2.0743	25.8418	42.4632	18.4542	64.7836	162.7340	263.4520	297.9030	129.9680	29.5038	26.9176
**CE(24:3)**	128.6980	156.1740	53.0350	60.2093	41.6874	91.8999	79.8557	93.5572	44.1379	153.0590	95.4858	612.0330	793.0340	401.0600	99.2142	53.0649
**CE(24:4)**	238.9120	286.7260	104.6940	93.3102	85.4382	180.3860	150.8630	123.9670	43.4823	152.9410	478.1820	668.7160	868.8380	723.9210	120.0990	70.3184
**CE(24:5)**	1393.7100	1577.6000	456.2170	459.0400	482.6170	1079.7600	943.2320	879.6760	239.8190	750.0420	2030.2800	3656.1000	4967.7300	3814.5300	639.2310	376.0420
**CE(24:6)**	1258.5100	1407.7400	252.1630	250.1270	285.1800	659.9860	694.9560	546.7470	115.8740	783.9800	2742.9200	5903.2900	8197.9600	3397.0600	564.6100	197.0310
**CE(25:1)**	36.4458	48.1008	15.1266	15.8942	9.5125	22.2198	30.7294	24.4729	11.4224	50.7554	125.3530	119.8110	156.1540	98.9595	21.4027	15.9591
**CE(25:3)**	20.7697	29.7337	9.5110	14.7466	5.9914	16.0815	16.4437	20.0612	4.5392	31.5259	97.3905	176.8370	220.2590	75.2442	14.3433	9.5059
**CE(25:4)**	19.1425	23.2932	11.9736	11.1386	11.9591	30.3109	21.0488	12.1315	3.3378	17.4018	19.6684	108.0660	125.3180	75.0639	9.0509	9.7027
**CE(26:0)**	8.1109	9.3854	3.9783	4.4923	5.8754	2.9895	5.7247	11.2969	11.0424	31.5172	68.3882	121.2370	130.2530	77.6220	11.8729	12.4895
**CE(26:1)**	120.0450	140.2680	38.0026	36.4151	7.8612	59.5440	56.3246	67.2757	50.2486	235.6000	18.4255	687.4200	886.6760	94.9100	81.1341	59.2224
**CE(26:2)**	66.8083	82.2106	25.1418	22.2771	16.7389	37.3133	38.2126	37.7067	19.1857	116.8690	6.6316	414.1550	467.3350	57.8683	54.3740	32.3937
**CE(26:3)**	161.2780	181.7990	63.8576	54.4639	38.0207	85.2274	86.2829	95.3494	51.3614	163.0300	33.7546	1168.3100	1341.1600	556.8660	88.6222	62.9678
**CE(26:4)**	144.2160	198.8150	56.1251	57.2380	34.0231	80.9391	98.1120	68.4562	37.4094	108.9180	367.8090	819.7570	940.7730	10.6333	70.5879	52.9291
**CE(26:5)**	412.3970	526.7460	112.3890	118.6730	113.9650	257.3260	259.3710	172.3250	54.0662	217.6150	155.6530	2489.2200	3251.4100	1335.6200	180.1730	88.1823
**CE(26:6)**	861.1450	966.5430	163.5110	164.5050	232.8230	532.0170	531.3550	368.4860	80.7519	375.8850	1479.0900	4782.8200	5895.5300	522.8120	297.4660	120.2150
**CE(27:1)**	12.5756	39.9602	15.1824	12.4333	9.1540	20.3750	24.1004	34.0551	23.6734	83.9308	147.6650	228.9930	232.9410	177.4840	23.0741	26.0281
**CE(27:2)**	15.0308	24.0372	3.5243	3.5342	1.2012	5.3191	9.4262	7.3862	5.3649	32.5565	82.8210	100.0140	114.4150	58.4937	7.1508	9.4992
**CE(28:1)**	18.2227	17.8383	6.2832	6.0154	4.0131	6.9367	8.4697	13.0958	14.6158	61.5514	142.3350	172.8840	193.8860	118.7870	15.3044	17.4321
**CE(28:2)**	7.7690	9.4531	1.8082	1.5850	0.3851	3.3089	5.7389	2.1082	5.2333	21.7693	72.9580	115.8680	133.4220	65.2896	4.6814	6.7579
**CE(28:3)**	13.9892	9.8295	4.7345	5.3547	4.3438	7.7845	7.5069	8.3865	6.7730	10.0608	56.1320	118.8950	146.8820	65.1184	8.5553	6.1893
**CE(28:6)**	51.8328	80.1431	8.7222	11.3439	1.1880	50.0286	27.9146	27.9523	6.7092	35.3432	1.6447	516.0020	664.6800	18.4379	4.9067	11.5266
**CE(30:1)**	3.8078	5.3007	3.2160	4.9018	2.2853	0.7458	2.6837	7.5057	5.8087	13.2495	0.3576	36.9998	47.9854	27.0479	6.5402	8.3207

*values represented as a ratio to internal standard

Supplementary Table S3

Supplementary Table S3. Raw data from the lipidomic analysis of cholesterol ester-fatty acid species in the brain tissue of WT and *Abcd1* KO mice.

Phenotype	WT 1	WT 2	WT 3	WT 4	WT 5	WT 6	KO 1	KO 2	KO 3	KO 4	KO 5	KO 6
Metabolite												
CE(14:0)	10.3896	11.3631	11.4830	14.0148	13.4745	12.2710	11.9395	11.1116	12.3518	12.1473	14.4328	14.4303
CE(15:0)	11.2798	8.1506	5.5674	9.2299	8.3177	10.5368	9.3632	7.3938	6.2209	7.4070	7.4758	9.9618
CE(16:0)	51.6107	51.8461	69.1106	60.0174	41.5529	68.5575	85.0761	42.5233	18.2893	36.1163	25.8172	41.7094
CE(16:1)	50.1504	59.5356	55.2129	57.8222	45.4364	65.1593	78.6044	42.4079	44.4836	59.3209	37.7132	53.3904
CE(16:2)	2.3677	1.5499	2.5066	1.4542	0.8992	1.2397	4.6116	0.5115	N/A	1.4465	1.1653	1.5388
CE(16:3)	5.8461	4.0196	7.3256	4.8367	2.6153	8.5842	8.8294	2.1079	0.7111	4.0882	1.2133	4.1912
CE(18:0)	7.0084	4.7955	6.0407	5.7356	2.3307	7.8519	9.5552	3.4416	1.5837	3.6742	3.1998	4.3084
CE(18:1)	577.5410	560.8730	629.5980	685.5360	438.2510	672.0250	944.7690	448.0770	295.3850	417.5380	352.3100	509.1570
CE(18:2)	1579.1400	1048.8200	1022.7900	1049.3300	1022.4700	1228.2500	721.1490	1030.3100	1291.5200	1344.0400	1416.5300	1873.9000
CE(18:3)	62.5835	48.5709	45.1199	54.0199	42.8631	51.0117	49.4175	45.3573	46.1130	57.8986	53.9265	73.3158
CE(19:0)	2.1290	1.5570	1.8614	2.1300	0.9266	2.2280	2.3480	0.8431	0.8132	1.0402	1.0791	1.5204
CE(19:1)	8.4155	8.4386	8.4111	10.4964	6.7760	11.7033	13.6277	6.0041	6.4709	6.3941	4.4636	7.4579
CE(19:2)	53.3448	25.7700	36.8873	27.0799	37.4484	50.9957	16.8785	45.5488	62.0210	38.4234	59.9996	71.6632
CE(19:3)	3.0074	1.3107	0.4909	2.3472	0.5668	3.0157	0.8107	3.0018	2.7826	3.4835	2.5361	5.7636
CE(20:1)	36.3656	19.1393	20.4307	25.8407	11.8522	28.0238	36.9964	13.9865	8.9497	13.9984	15.2267	24.4213
CE(20:2)	53.0685	33.5653	44.5556	37.5074	28.7605	38.6040	41.5531	35.1556	27.7141	26.2015	3.2962	42.2372
CE(20:3)	197.7690	147.2700	197.6270	185.5930	119.7150	220.6830	207.5020	116.2020	117.6830	172.1020	118.5290	167.5510
CE(20:4)	2317.2800	2171.2000	2089.3300	2516.9700	1663.0400	2152.4700	1860.5300	1761.1900	1873.8700	2338.9900	2107.1300	2801.8500
CE(20:5)	131.2510	108.1180	141.3140	121.3570	79.4226	155.1610	143.3470	76.4806	74.1930	111.4340	60.1420	116.1000
CE(21:1)	1.7646	0.9787	0.9581	1.4427	0.4608	1.5066	1.5194	0.7598	0.5834	0.5831	0.9522	1.1151
CE(21:3)	3.3638	2.4399	3.1950	2.9537	1.9047	2.8167	2.9151	1.5255	1.7279	3.4317	2.4516	3.0376
CE(21:4)	56.9262	50.1348	44.4128	46.2023	45.4795	56.7508	39.2479	50.0843	56.0943	48.3840	59.1500	74.7431
CE(21:5)	5.4733	4.0287	4.7282	1.6743	3.5304	6.0490	1.8986	3.6957	4.2325	2.2027	1.9579	6.4666
CE(22:1)	5.5100	2.9651	3.5119	3.9671	2.5443	3.9464	3.1145	2.8338	2.8070	3.4288	4.1517	5.2125
CE(22:2)	3.3779	1.7415	2.0820	1.8264	1.3561	2.1185	2.2360	1.9959	1.0300	2.0699	2.2443	2.3954
CE(22:3)	12.5502	6.3111	7.5512	5.9426	5.9912	8.4303	8.8591	6.1244	4.9815	6.2717	6.3847	7.8028
CE(22:4)	235.2930	193.5790	260.6240	252.8610	147.7660	259.7860	374.5510	148.4760	102.4840	157.1450	118.6380	174.6250
CE(22:5)	230.3570	147.3560	207.8000	184.6500	87.0441	246.4860	265.2060	89.1001	56.6415	127.5830	89.4179	179.7920
CE(22:6)	3041.1300	2432.5700	3547.1800	3126.4100	1477.3500	3783.0300	3754.2400	1653.7800	879.3450	1974.2600	1657.4500	2697.4300
CE(23:1)	2.3906	1.3912	1.0820	1.4934	0.6538	1.4895	1.5085	1.1638	0.7861	1.1062	1.3472	1.7897
CE(23:5)	3.4448	2.2507	3.4172	2.1710	1.5172	4.0437	4.2455	1.9107	1.2253	1.5161	1.0499	3.6366
CE(23:6)	35.0235	31.2719	43.8801	35.9008	22.4231	47.0751	45.6818	24.7624	15.7660	23.6807	23.4354	39.1349
CE(24:0)	0.3987	0.3778	0.4836	0.2155	0.3837	0.2903	0.1614	0.6358	0.7758	1.0334	0.8296	0.9931
CE(24:1)	7.7472	6.0774	5.7759	5.5071	4.2469	6.1497	6.0031	7.0594	7.2524	9.3927	10.8746	11.6959
CE(24:2)	1.3816	0.7148	1.2218	1.0038	0.7836	1.0063	1.1625	1.4300	1.1804	1.4735	1.8380	2.4464
CE(24:3)	1.2239	0.8386	0.8306	0.9378	0.8684	1.1079	0.8489	0.7088	0.6306	1.1703	1.2232	1.5675
CE(24:4)	21.6194	8.5440	13.0893	8.9330	6.4961	17.7696	22.9491	5.9468	4.1163	8.7633	6.9574	10.4619
CE(24:5)	45.8774	31.6606	55.7363	38.2021	21.8871	56.3519	86.6082	21.2911	11.3484	28.3760	19.3021	29.9169
CE(24:6)	63.2579	39.7367	64.8949	55.4578	24.2084	78.3374	90.6438	24.9807	11.0362	27.8175	23.2143	44.1073
CE(25:1)	0.7081	0.4790	1.0899	0.3498	0.4141	0.6665	0.8074	1.0677	1.2913	1.2497	2.6928	0.8934
CE(25:4)	1.0781	0.5653	0.7537	0.6149	0.5663	0.8475	0.8817	0.4050	0.4402	0.7538	0.7362	0.8194
CE(26:0)	0.1532	0.0413	0.0462	0.1140	0.1347	0.1478	0.5924	1.0440	0.5432	0.9867	0.6468	1.1108
CE(26:1)	1.4324	0.7870	0.4081	0.5225	0.8056	1.2949	3.6321	2.5332	2.6545	4.1237	3.3585	4.1743
CE(26:2)	0.2014	0.1287	0.1517	N/A	N/A	0.0956	0.3188	0.6823	0.2984	0.4124	0.3209	0.9520
CE(26:4)	1.3437	1.1130	1.0202	0.9458	0.7178	1.1145	1.1510	1.1919	0.6923	0.9639	1.3083	1.3954
CE(26:5)	2.0068	1.1178	1.4104	1.0687	0.9380	1.5670	2.1504	0.7880	0.7374	1.0804	0.9493	1.1178
CE(26:6)	4.4405	2.4083	4.2152	2.7143	1.4502	4.7479	5.4805	1.7011	0.8145	2.2622	1.6666	2.8137

*values represented as a ratio to internal standard

Supplementary Table S4

Supplementary Table S4. Raw data from the lipidomic analysis of cholesterol ester-fatty acid species in the spinal cord tissue of WT and *Abcd1* KO mice.

**Phenotype**	**WT 1**	**WT 2**	**WT 3**	**WT 4**	**WT 5**	**WT 6**	**KO 1**	**KO 2**	**KO 3**	**KO 4**	**KO 5**	**KO 6**
**Metabolite**												
**CE(14:0)**	5.9504	5.0152	6.7368	7.8825	3.5090	6.3873	6.7238	7.6141	9.8397	5.3606	2.7932	4.1618
**CE(15:0)**	14.3717	8.7176	4.7923	11.6867	11.1615	18.1543	12.8208	16.2987	19.2521	13.1559	7.5366	16.3191
**CE(16:0)**	9.1871	4.1887	10.0442	4.9257	8.8098	8.2684	4.9875	6.3290	3.4324	5.0627	8.2601	10.2674
**CE(16:1)**	15.6930	23.6007	19.9298	15.3252	26.1694	16.4050	23.8388	14.9623	14.9696	16.4789	17.1327	33.2011
**CE(16:2)**	N/A	N/A	0.4558	N/A	N/A	N/A	0.2703	N/A	N/A	0.4943	N/A	N/A
**CE(16:3)**	N/A	N/A	N/A	0.3185	N/A	N/A	N/A	N/A	N/A	N/A	N/A	N/A
**CE(18:0)**	1.2217	1.3270	1.5841	0.9321	1.7382	1.0751	1.1301	1.3642	1.7259	1.4061	2.3027	1.1878
**CE(18:1)**	146.5850	182.1690	170.9200	131.5080	213.3400	147.7060	175.0200	164.3300	157.7260	153.3260	197.8830	238.7280
**CE(18:2)**	1085.5500	1028.0900	842.3400	670.0250	1241.9300	755.5030	897.3150	1103.4000	914.5760	444.4670	947.9890	1507.0100
**CE(18:3)**	35.1683	36.9514	27.3067	29.9680	37.1951	22.3762	36.5819	36.7779	32.6668	20.1315	28.9013	48.8367
**CE(19:0)**	N/A	0.1458	0.2258	0.0482	0.3256	0.0602	N/A	0.0802	N/A	0.0617	0.0678	0.2119
**CE(19:1)**	2.2938	2.5345	2.8584	2.0908	2.9931	1.2502	1.3104	2.0974	1.5004	2.4600	2.1333	5.1387
**CE(19:2)**	39.8477	30.5277	24.0948	14.6207	38.0690	24.6644	15.6756	33.2062	35.3707	6.7195	30.8246	47.4538
**CE(19:3)**	1.7553	0.5807	0.2058	0.2458	0.7767	0.5553	0.8009	1.4926	1.4714	0.1815	0.5039	1.4874
**CE(20:1)**	7.8295	8.4759	8.8817	7.1060	15.3938	7.4768	8.5927	10.5896	13.3771	7.6493	12.9788	13.8401
**CE(20:2)**	15.9201	13.1074	18.5733	9.5564	27.4564	13.9907	15.6086	22.0694	16.0441	9.4487	23.2823	28.2443
**CE(20:3)**	61.7535	74.4036	68.1342	51.9723	77.5587	56.8083	76.7994	59.6645	52.2163	43.4943	72.5810	104.9680
**CE(20:4)**	1005.2000	1406.2700	1171.7200	1088.1400	1159.2900	970.5770	1163.1200	1283.7300	1052.3900	857.0750	960.2780	1268.7600
**CE(20:5)**	26.5986	19.5191	25.0113	24.3055	20.4488	19.9293	48.2339	27.8801	27.5347	14.5068	17.9909	56.2040
**CE(21:1)**	0.0760	0.3279	0.3354	0.0604	0.5366	0.0797	N/A	0.3596	0.4648	N/A	0.0584	0.1369
**CE(21:3)**	0.6811	0.3023	0.3257	0.8679	0.7605	0.4028	0.7865	0.4900	N/A	0.2985	0.7330	1.0961
**CE(21:4)**	23.5953	27.6409	21.0684	13.6732	20.5537	20.2071	19.1855	29.6505	27.1177	10.1917	17.2323	30.5437
**CE(21:5)**	1.7154	1.0323	0.7366	0.8237	1.0219	0.6302	0.4191	2.3838	1.3189	N/A	0.6164	2.2291
**CE(22:1)**	3.1827	2.8923	3.1343	2.9939	4.6947	3.3544	4.5978	4.9030	5.6044	3.3027	4.4465	5.5671
**CE(22:2)**	0.8829	0.4334	1.1419	0.4649	1.1128	0.5487	1.2192	1.5425	0.7028	0.9012	1.4094	2.2677
**CE(22:3)**	3.2260	2.3567	3.0051	1.6291	3.2024	1.8190	3.0201	4.0883	2.8258	3.0016	4.5087	5.5808
**CE(22:4)**	57.5461	49.3771	70.0682	34.5320	58.9636	38.1218	53.5838	66.9497	62.3615	64.1496	76.8703	77.9020
**CE(22:5)**	21.9684	24.0891	21.8576	15.6577	28.3142	14.5395	25.3514	25.3150	20.0170	19.9225	24.3967	37.3894
**CE(22:6)**	398.0030	469.8940	424.6530	423.1130	414.7590	348.3750	424.6650	478.2510	382.2320	341.0420	352.0940	508.7500
**CE(23:1)**	0.6277	0.0999	0.5516	0.6508	0.8039	0.3093	0.8963	0.5881	0.7243	0.6741	1.2031	1.5972
**CE(23:5)**	N/A	N/A	0.1078	N/A	N/A	N/A	0.1272	N/A	N/A	N/A	N/A	N/A
**CE(23:6)**	4.5600	6.5550	3.2380	2.4650	1.3360	4.3882	2.7293	6.0165	5.1973	2.9893	1.2932	8.2609
**CE(24:0)**	0.4261	0.3676	0.4942	0.1286	0.6137	0.3355	0.9491	0.7251	0.4126	0.1918	0.6243	1.1065
**CE(24:1)**	6.7753	3.5589	5.9907	4.5060	7.3649	4.6681	11.0791	12.3482	10.1131	7.6391	9.8719	11.6240
**CE(24:2)**	1.2226	0.3597	1.0866	0.2762	1.0436	0.2489	2.2061	1.5712	0.7705	0.8660	1.6381	2.5888
**CE(24:3)**	0.6358	0.3030	0.6132	0.2529	0.2880	0.2448	0.8873	1.1882	0.0948	0.4769	0.6371	1.1553
**CE(24:4)**	3.9980	2.9632	5.4125	1.4373	2.9440	1.8735	3.6919	5.8766	3.0569	4.0344	5.8932	5.8673
**CE(24:5)**	1.0307	2.1384	3.1268	1.7107	3.4732	2.2212	2.2683	3.7189	1.6950	2.7508	2.2538	4.3881
**CE(24:6)**	4.7606	3.8379	3.2384	3.0072	4.5302	3.1389	2.9873	4.8540	2.6163	3.1658	5.1096	5.0074
**CE(25:1)**	0.1621	0.2701	0.2771	0.3115	0.5517	0.0523	1.2435	1.3205	0.6002	1.0615	0.8969	0.6468
**CE(25:4)**	0.2182	0.4568	0.2392	0.1328	0.3503	0.3265	0.3727	0.3371	0.1146	1.0319	0.3598	0.2397
**CE(26:0)**	N/A	0.0694	N/A	0.1127	0.1699	N/A	0.6976	0.7261	0.2993	0.2046	1.1719	0.3894
**CE(26:1)**	0.7648	0.7163	0.7904	0.8990	0.8869	0.7655	4.0332	3.9178	3.2868	2.7386	3.4363	3.6231
**CE(26:2)**	0.0803	0.0643	0.1360	N/A	N/A	0.1460	0.5035	0.9271	0.0830	0.4261	0.7066	0.3618
**CE(26:4)**	0.8622	0.5844	0.7408	0.1300	0.1938	0.0811	1.5889	1.7319	0.6018	1.6570	1.8949	1.0269
**CE(26:5)**	0.7313	0.1961	0.4168	N/A	0.4146	0.1204	0.6610	0.5565	N/A	0.6094	1.1240	0.7271
**CE(26:6)**	0.1527	0.3923	0.2660	0.0956	0.1167	0.1523	0.2120	0.2449	N/A	0.1693	0.1146	0.4692

*values represented as a ratio to internal standard

Supplementary Table S5

Supplementary Table S5. *P*-values of *t*-tests comparing the effect of cholesterol condition on gene expression within the control and X-ALD genotype groups.

**Gene**	**LDM *vs* complete medium***	**LDM *vs* LDM+chol***	**Complete medium *vs* LDM+chol ***
**Control fibroblasts**			
HMGCR	p<0.0001	p<0.0001	p=0.0050
SOAT1	p=0.0767	p=0.0029	p=0.0637
NCEH1	p=0.4960	p=0.3147	p=0.7375
ABCA1	p=0.0045	p=0.0005	p=0.0013
**X-ALD fibroblasts**			
HMGCR	p=0.0023	p=0.0009	p=0,0001
SOAT1	p=0.9731	p=0.5754	p=0.4919
NCEH1	p=0.5367	p=0.8286	p=0.7155
ABCA1	p=0.0065	p=0.0020	p=0.0014

* Complete medium, standard RPMI with 10% FBS; LDM, RPMI with 10% lipid-depleted FBS; LDM+chol, LDM with 10 µg/ml cholesterol

Supplementary Figure S1

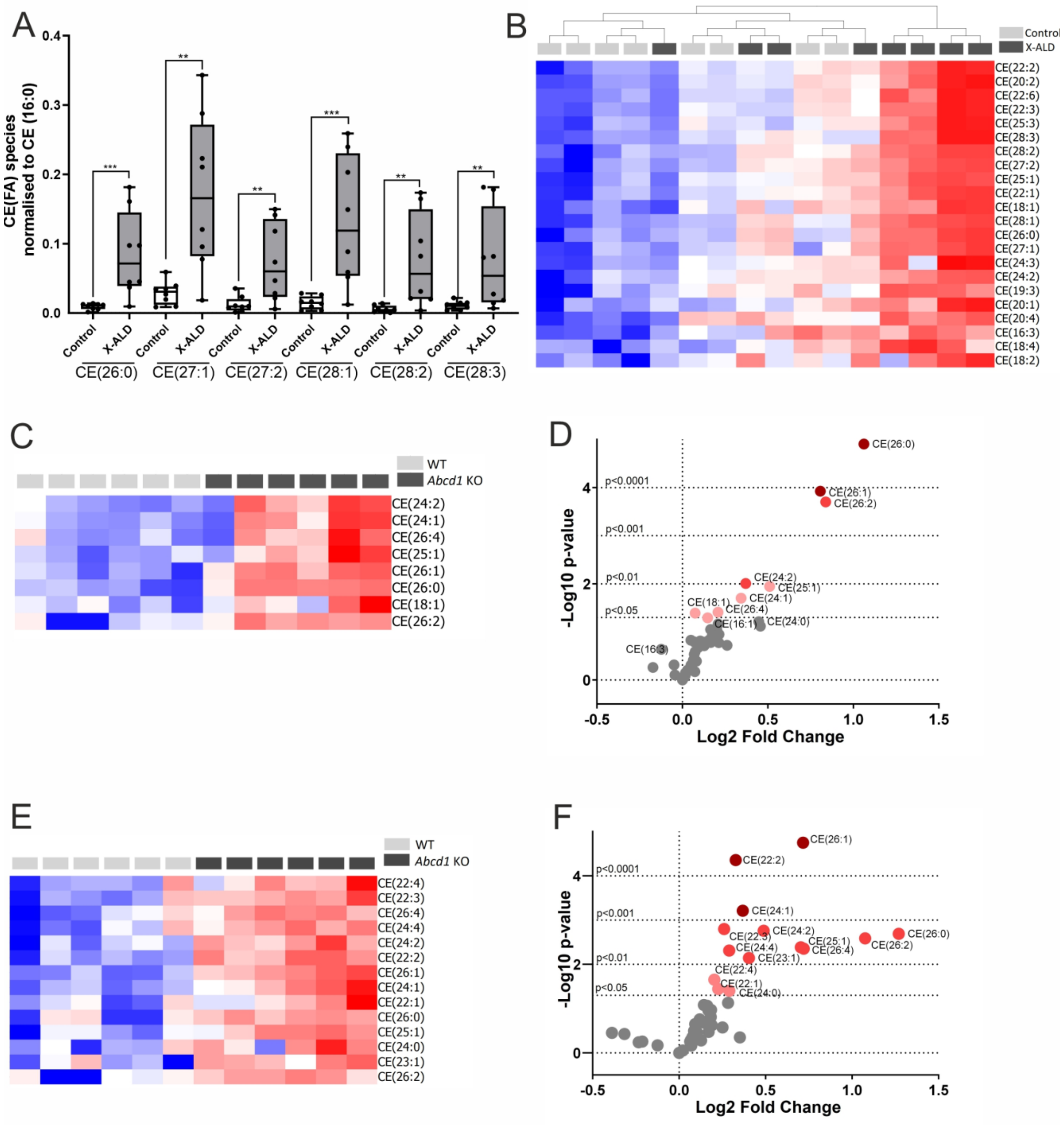

Supplementary Figure S1. Lipidomic analysis of cholesterol ester-fatty acid species in human control and X-ALD fibroblasts and in the CNS of WT and *Abcd1* KO mice. (A) Relative levels of the top six significantly altered CE(FA)s normalised to CE(16:0) and **(B)** heat map depicting all significantly different CE(FA)s in X-ALD *vs* control fibroblasts (*n*=8 each). **(C)** Heat map and **(D)** volcano plot depicting significantly different CE(FA)s in the brain of *Abcd1* KO *vs* WT mice (*n*=6 each). **(E)** Heat map and **(F)** volcano plot depicting significantly different CE(FA)s in the spinal cord of *Abcd1* KO *vs* WT mice (*n*=6 each). Qlucore Omics Explorer 3.5 software was used to analyse the data and generate the heat maps. *P*-values and log2-fold change values obtained from the Qlucore software (after performing two group comparison test) were used to create volcano plots in GraphPad Prism 8. * $p < 0.05$; ** $p < 0.01$; *** $p < 0.001$.

Supplementary Figure S2

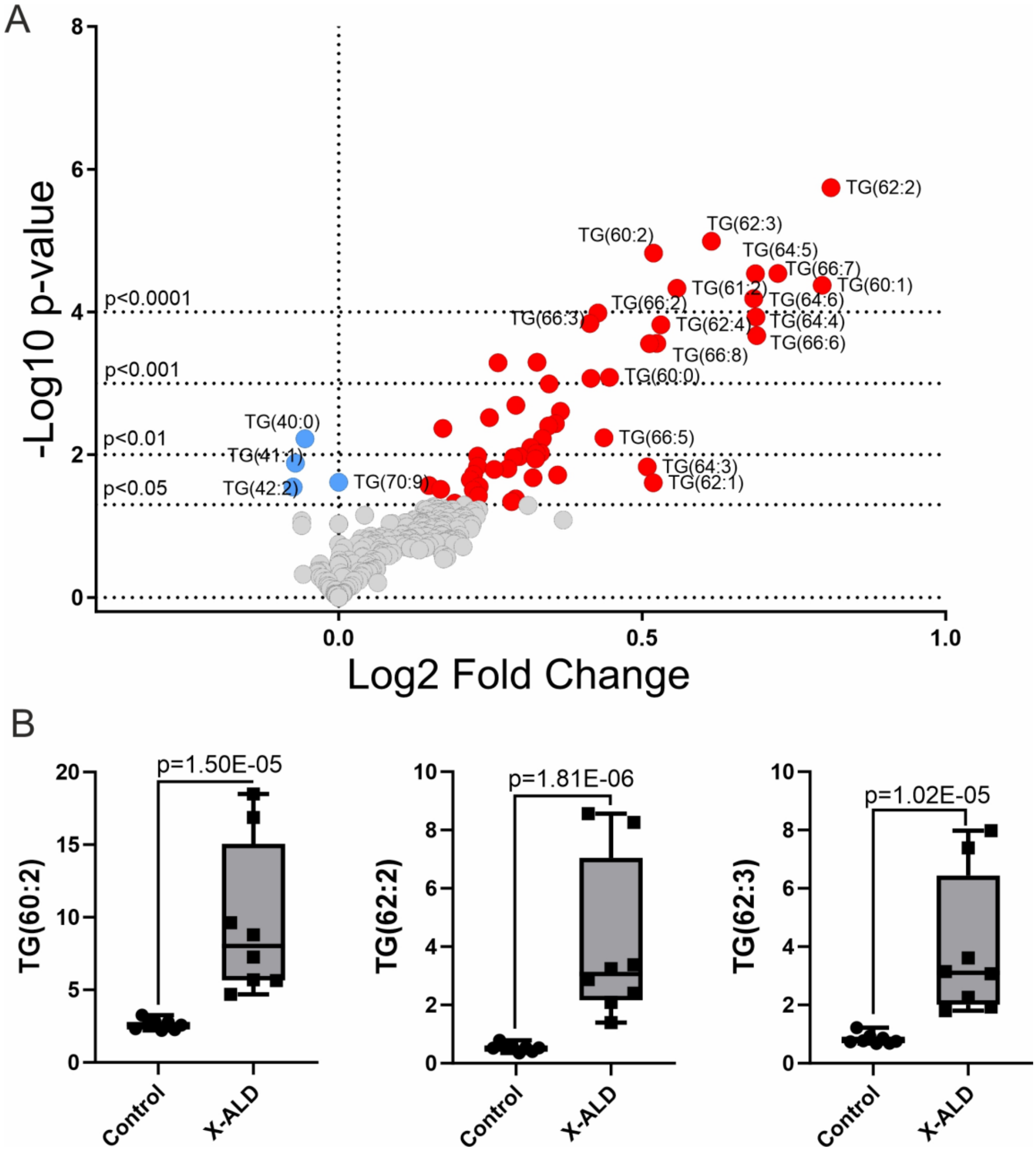

Supplementary Figure S2. Lipidomic analysis of triglyceride-fatty acid species in human control and X-ALD fibroblasts. (A) Volcano plot depicting significantly different TG(FA)s (normalised to the internal standard) between control and X-ALD fibroblasts (*n*=8 each). **(B)** Top three significantly altered TG(FA) in X-ALD *vs* control fibroblasts (*n*=8 each). In (B), values represent the ratios to the internal standard. Qlucore Omics Explorer 3.5 software was used to analyse the data. *P*-values and/or log2-fold change values obtained from the Qlucore software (after performing a two-group comparison test) were used to create volcano plots in GraphPad Prism 8.

Supplementary Figure S3

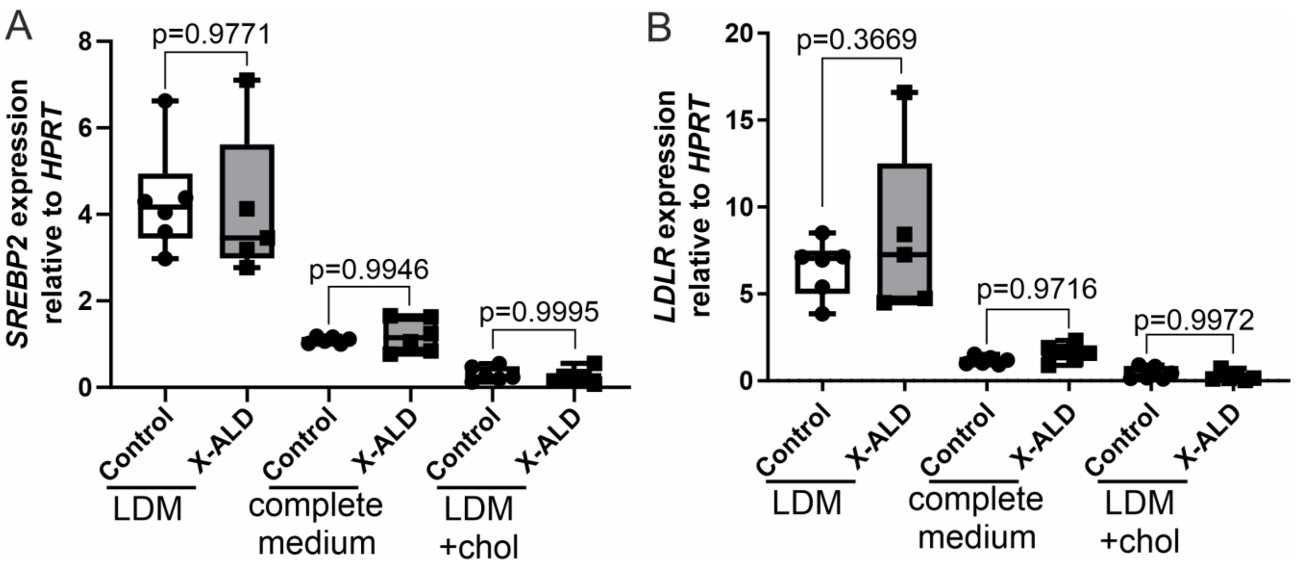

Supplementary Figure S3. Cholesterol triggers proper regulation of the SREBP2 pathway in control and X-ALD fibroblasts at the mRNA level. (A, B) Primary fibroblasts derived from healthy controls or X-ALD patients (*n*=6 each) were cultivated in lipid-depleted medium (LDM), complete RPMI medium or LDM supplemented with 10 µg/ml cholesterol for 5 days (medium was refreshed after 48 h). The mRNA levels of *SREBP2* **(A)** and its transcriptional target, *LDLR* (**B**), were determined by RT-qPCR relative to *HPRT*. The data are depicted as box plots displaying all values and the median. One-way ANOVA with Sidak's multiple comparisons test was performed for statistical analysis of the data.

Supplementary Figure S4

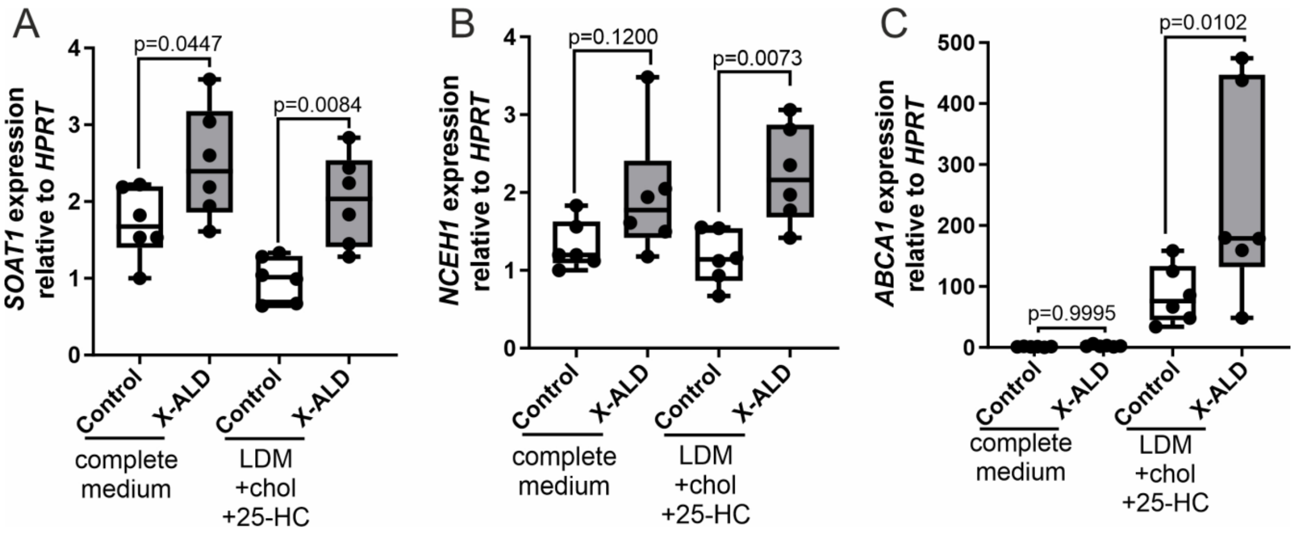

Supplementary Figure S4. Dysregulated cholesterol-associated gene expression in X-ALD is aggravated by combined cholesterol and 25-HC exposure. (A-C) Primary fibroblasts derived from healthy controls or X-ALD patients (*n*=6 each) were cultivated in complete RPMI medium or lipid-depleted medium (LDM) supplemented with 10 µg/ml cholesterol and 1 µg/ml 25-hydroxy cholesterol (25-HC) for 5 days (medium was refreshed after 48 h). The mRNA levels of *SOAT1* **(A)**, *NCEH1* **(B)** and *ABCA1* **(C)** were determined by RT-qPCR relative to *HPRT*. The data are depicted as box plots displaying all values and the median. One-way ANOVA with Sidak's multiple comparisons test was performed for statistical analysis of the data.

Supplementary Figure S5

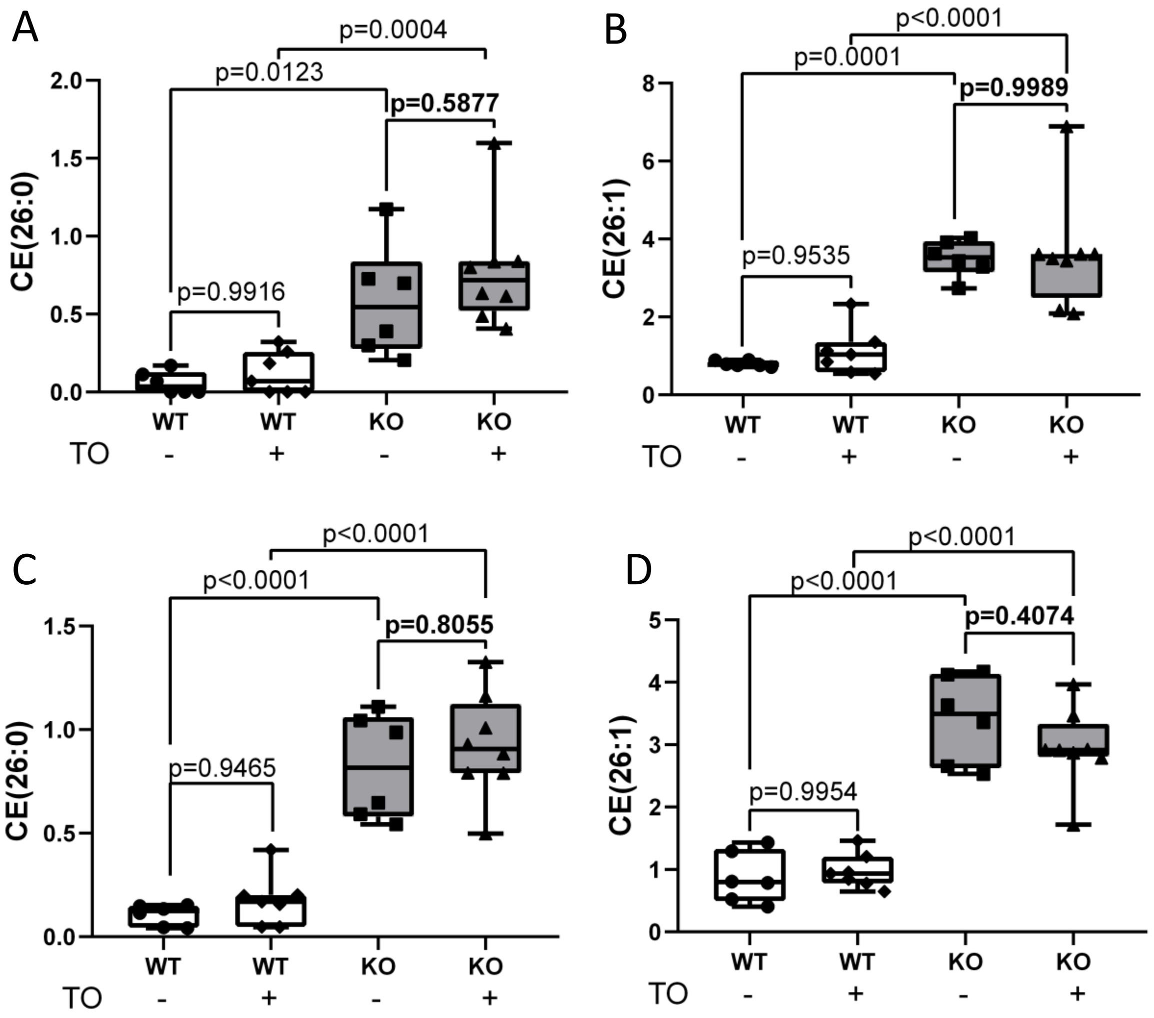

Supplementary Figure S5. LXR agonist TO901317 does not reduce the levels of the most significantly accumulating CE-VLCFA species, CE(26:0) and CE(26:1), in the CNS of X-ALD mice. (A-D) WT (*n*=7) and *Abcd1* KO (*n*=8) mice received chow supplemented with 100 mg/kg TO901317 (TO) for 10 weeks; alternatively, WT (*n*=6) and *Abcd1* KO (*n*=6) were fed a normal diet. At the treatment endpoint, mice were sacrificed for lipidomic analysis of the brain **(A)** CE(26:0) and **(B)** CE(26:1) levels in the spinal cord and **(C)** CE(26:0) and **(D)** CE(26:1) levels in brain tissue of TO treated or untreated WT *Abcd1* KO mice. The data are depicted as box plots displaying all values and the median. One-way ANOVA with Sidak's multiple comparisons test was performed for statistical analysis of the data.

Supplementary Figure S6

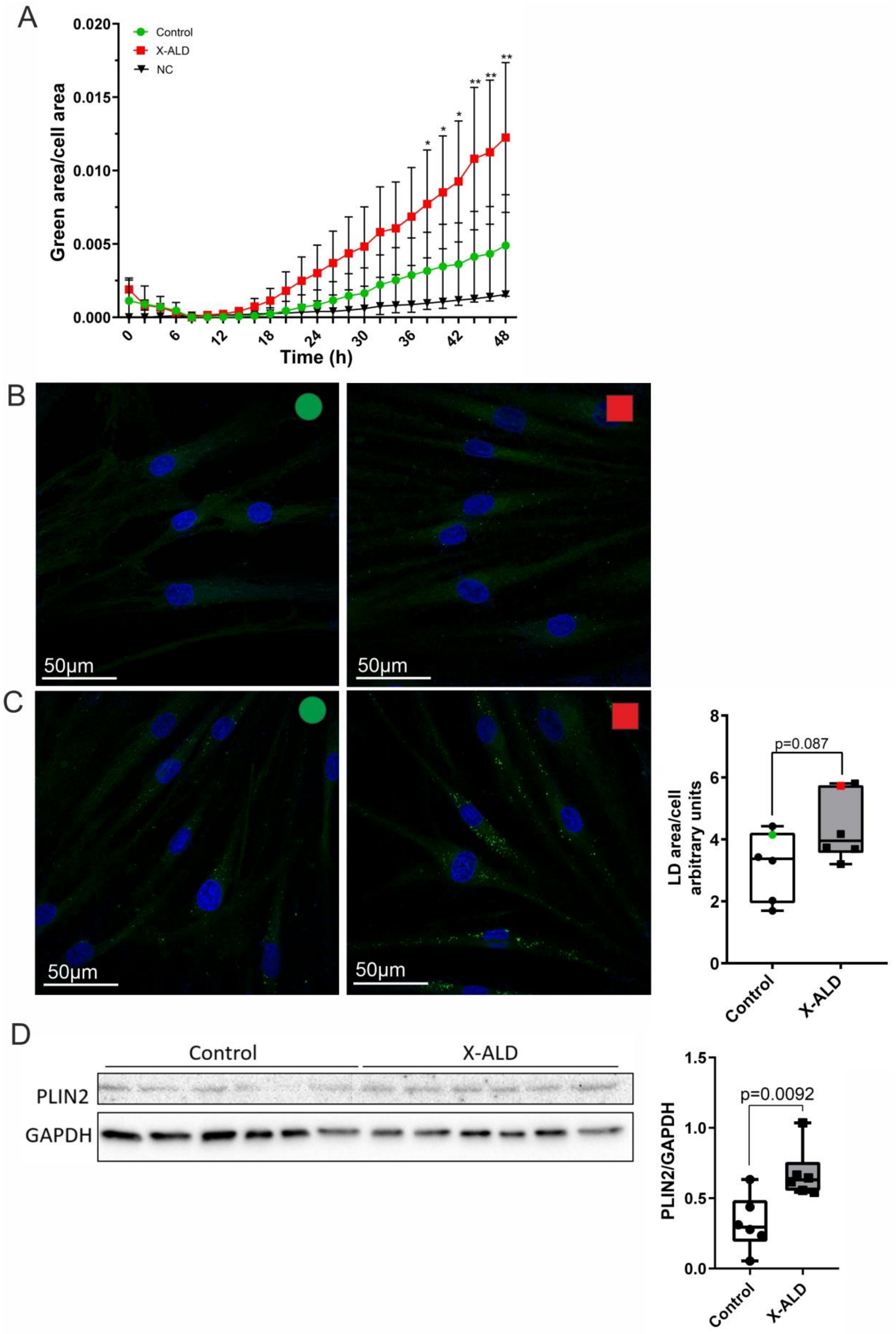

Supplementary Figure S6. Increased induction of LDs is manifest in X-ALD fibroblasts already after 24 h of cholesterol loading. (A–D) Control and X-ALD-derived primary human (*n*=6 each) fibroblasts were starved for 72 h in lipid-depleted medium (LDM) and then incubated for 24 or 48 h with 20 µg/ml cholesterol (in LDM). **(A)** The time course of cholesterol–induced LD formation was measured by using the neutral lipid stain BODIPY™ 493/503 and Incucyte® live imaging. Results are expressed as green fluorescent area/cell area. NC, normal control fibroblast line cultured in LDM with BODIPY™ 493/503 and EtOH (vehicle). Confocal microscopy pictures of control (left panel) and X-ALD (middle panel) cells cultured in LDM for 72 h **(B),** treated next for 24 h with 20 µg/ml cholesterol **(C)** and stained with BODIPY™ 493/503 (LDs, green fluorescence) and DAPI (nuclei, blue). The summary statistics (right panel) show the results as LD area per cell. The data points for the samples shown to the left are colour-coded in the box plots. **(D)** Immunoblot analysis of perilipin 2 (PLIN2) protein expression after 24 h of 20 µg/ml cholesterol treatment. The relative levels of PLIN2 normalised to GAPDH are displayed to the right. In A, the data are depicted as mean ± SD; two-way ANOVA with Dunnett's multiple comparisons test (* $p < 0.05$; ** $p < 0.01$). In C and D, the box plots show all values and the median; unpaired two-sided Student's t-test.

Supplementary Figure S7

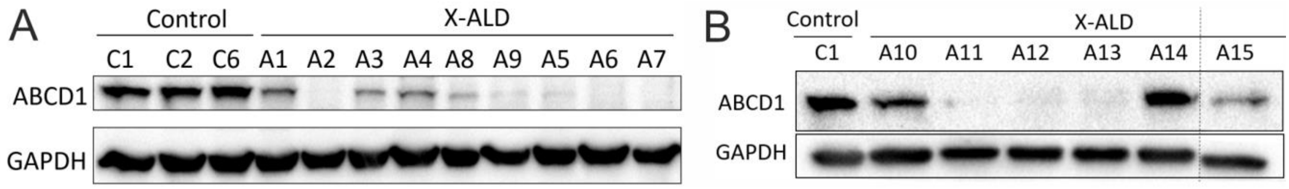

Supplementary Figure S7. ABCD1 protein levels in various X-ALD-derived fibroblast lines. Representative immunoblots of ABCD1 protein and, for normalisation, GAPDH expression in three control and 15 X-ALD primary fibroblast lines (**A**, **B**). A dashed line indicates a cut in the image.

Supplementary Figure S8

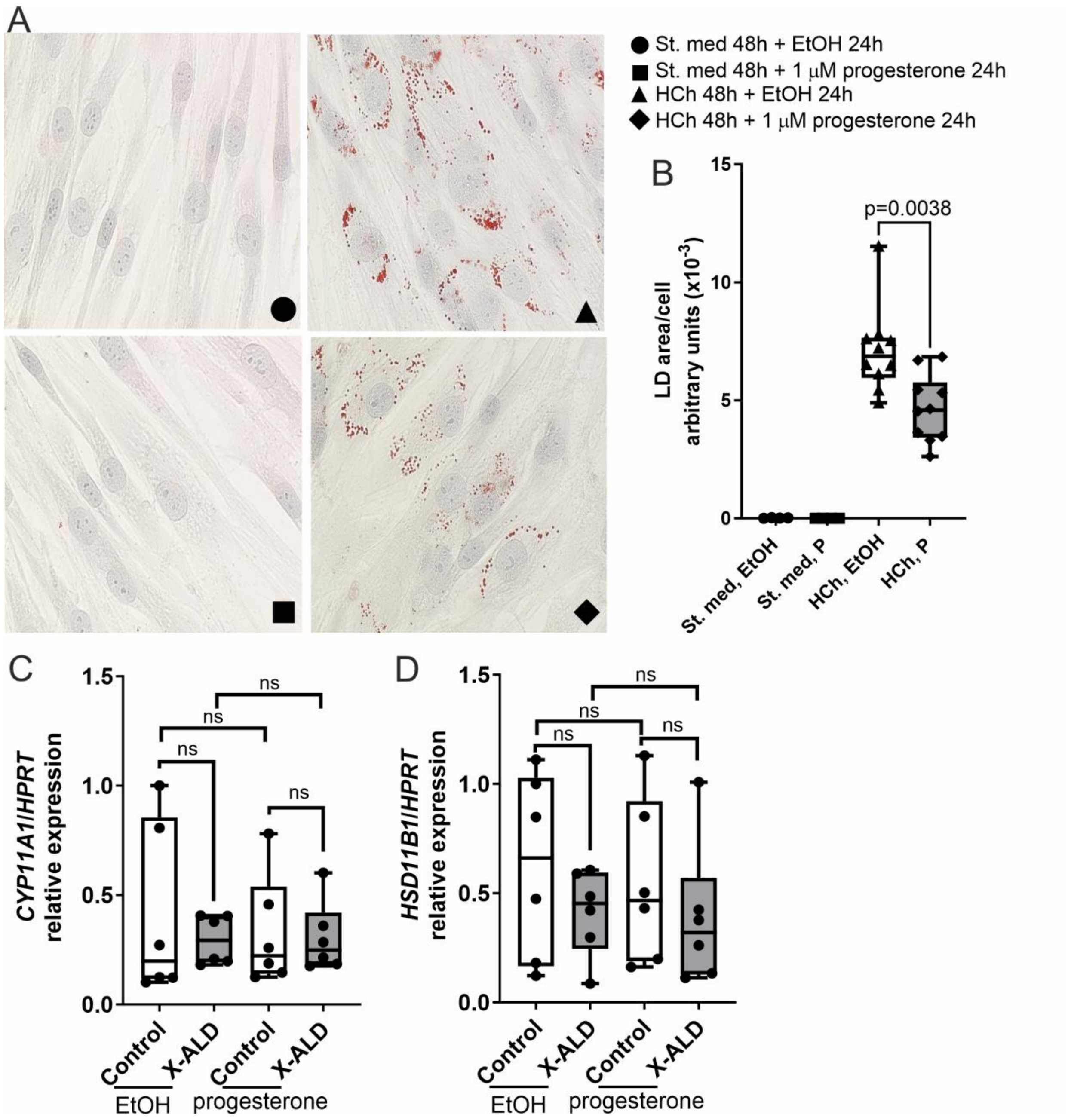

Supplementary Figure S8. Progesterone triggers LD lipolysis in fibroblasts without affecting the expression of genes associated with the cortisol pathway. (A, B) A human control fibroblast line was cultured for 48 h in a complete RPMI medium (St. med) or LDM supplemented with 20 µg/ml cholesterol (HCh) to induce LDs. Subsequently, cells were treated for 24 h with 1 µM progesterone (P) or vehicle (EtOH) followed by ORO staining to visualise LDs. **(A)** Representative light microscopy pictures of ORO-stained cells from all culture conditions. **(B)** For quantification of the cellular LD content, ten randomly chosen pictures were analysed per condition and plotted as stained area per cell. **(C, D)** Control and X-ALD fibroblasts (*n*=6 each) in complete RPMI medium were treated for 24 h with 1 µM progesterone or vehicle (EtOH) before harvesting the cells for RT-qPCR analysis of gene expression involved in the cortisol pathway. The relative mRNA levels of *CYP11A* **(C)** and *HSD11B1* **(D)** were normalised to that of *HPRT*. In B-D, the box plots show all values and the median, one-way ANOVA with Tukey's multiple comparison test.
